# Response to “Leigh-like syndrome with mild mtDNA depletion due to the SUCLG1 variant c.626C>A”

**DOI:** 10.1016/j.ymgmr.2018.12.002

**Published:** 2018-12-13

**Authors:** Christos Chinopoulos, Ron A. Wevers, Hans R. Waterham, Dimitrios Zafeiriou

**Affiliations:** a Department of Medical Biochemistry, Semmelweis University, Budapest, Hungary; b Translational Metabolic Laboratory, Department Laboratory Medicine, Radboud University Medical Centre, Nijmegen, The Netherlands; c Laboratory Genetic Metabolic Diseases, Amsterdam University Medical Centers, University of Amsterdam, The Netherlands; d 1st Department of Pediatrics, “Hippokratio” General Hospital, Aristotle University, Thessaloniki, Greece

We thank Dr. Finsterer for his interest [1] in our work [2].

The patient presented initially with generalized hypotonia and exaggerated tendon reflexes and progressively developed spasticity and dystonia. This is not uncommon in cases of pyramidal tract involvement of central origin and is often seen in premature-born babies with periventricular leucomalacia, first exhibiting a variable degree of hypotonia and later on develop hypertonia (*i.e.* spasticity).

Regarding the twin sister, no autopsy was performed, thus no information can be obtained as of her phenotypic or genotypic features and if an mtDNA depletion syndrome was involved.

Regarding respiratory chain complex functions, we considered that the overall respiratory chain activity of the patient fibroblasts is normal because when using glucose, oxygen consumption rates were not different from those of controls (figure panels 3C and 4E in [2]). Furthermore, baseline mitochondrial membrane potential (ΔΨm) values during state 2 and state 3 respiration normalized to maximal depolarization caused by the uncoupler SF6847 (Fig. 2, before and after addition of ADP, respectively), were also not different. This means that the changes in individual respiratory complex activities –if any- didn't confer a sufficient damage for impairing overall respiratory chain function. This is important, because we could not compare respiratory chain component activities of the patient fibroblasts (i.e. n = 1) to three controls using a sufficiently robust statistical test.

Regarding the possibility of greater mtDNA depletion and/or respiratory chain complex dysfunctions in severely affected tissues such as the brain, we reiterate what we wrote in the manuscript, namely that this may well be possible. Having said that, we emphasize that we do not support pursuing brain sampling at autopsy; as this is performed only rarely for a number of reasons and in view of the fact of the rarity of the disease itself, a sufficient number of patients for mtDNA and respiratory chain complex function determinations may take decades to pool. Instead, we underscore that mtDNA and respiratory chain complex function determinations from fibroblasts does not reflect the severity of the disease in other tissues; however, oxygen consumption rates using glutamine instead of glucose, imaging of mitochondrial morphology and measuring mitochondrial substrate-level phosphorylation in cultured skin fibroblasts do provide definite answers regarding the impact of succinate-CoA ligase deficiency on mitochondrial bioenergetics, rendering brain autopsy procedures obsolete.

Regarding more conclusively explaining the reduction and mislocalization of SUCLG2 protein in view of the mutation in *SUCLG1* gene, this finding is indeed worth pursuing on its own. Mindful that to the best of our knowledge, known molecular biology/signal transduction pathways cannot offer a suitable insight and because the cultured fibroblasts of the deceased patient will exhibit senescence after a defined number of divisions, experiments to address this observation are planned so as to minimize the chance of running out of this crucial biological material.

Concerning the dissimilar phenotype between the father and the daughter despite the same genotype, at the cDNA level the c.626C > A mutation was found to be homozygous and therefore we described the patient as functionally homozygous for this mutation [2]. The maternal allele was not expressed and the underlying cause has not been identified. Multiplex ligation-dependent probe amplification excluded the possibility of a larger deletion. Further analysis of the cause for not expressing the maternal allele would not only require analyses of the *SUCLG1* introns but also a search for a pathogenic mutation in the promotor regions of this gene or in the 3′ untranslated region (UTR) of the gene that can cause allelic expression imbalance [3].
